# Moderators of the longitudinal relationship between the perceived physical environment and outside play in children: the KOALA birth cohort study

**DOI:** 10.1186/s12966-014-0150-8

**Published:** 2014-12-12

**Authors:** Teun Remmers, Dave Van Kann, Jessica Gubbels, Swantje Schmidt, Sanne de Vries, Dick Ettema, Stef PJ Kremers, Carel Thijs

**Affiliations:** Maastricht University (Medical Center+), CAPHRI School for Public Health and Primary Care, Department of Epidemiology, P.O. Box 616, 6200MD Maastricht, The Netherlands; Maastricht University (Medical Center+), CAPHRI School for Public Health and Primary Care, Department of Health Promotion, Maastricht, The Netherlands; Maastricht University (Medical Center+), NUTRIM School for Nutrition, Toxicology and Metabolism, Department of Health Promotion, Maastricht, The Netherlands; TNO Department of Life Style, Leiden, The Netherlands; The Hague University of Applied Sciences, The Hague, The Netherlands; Department of Social Geography, Utrecht University, Utrecht, The Netherlands

**Keywords:** Moderation, Interaction, Perceived physical environment, Physical activity, Parenting, Social environment

## Abstract

**Objectives:**

Promoting unstructured outside play is a promising vehicle to increase children’s physical activity (PA). This study investigates if factors of the social environment moderate the relationship between the perceived physical environment and outside play.

**Study design:**

1875 parents from the KOALA Birth Cohort Study reported on their child’s outside play around age five years, and 1516 parents around age seven years. Linear mixed model analyses were performed to evaluate (moderating) relationships among factors of the social environment (parenting influences and social capital), the perceived physical environment, and outside play at age five and seven. Season was entered as a random factor in these analyses.

**Results:**

Accessibility of PA facilities, positive parental attitude towards PA and social capital were associated with more outside play, while parental concern and restriction of screen time were related with less outside play. We found two significant interactions; both involving parent perceived responsibility towards child PA participation.

**Conclusion:**

Although we found a limited number of interactions, this study demonstrated that the impact of the perceived physical environment may differ across levels of parent responsibility.

**Electronic supplementary material:**

The online version of this article (doi:10.1186/s12966-014-0150-8) contains supplementary material, which is available to authorized users.

## Background

Physical activity (PA) is key to prevent and reverse childhood overweight and obesity, resulting in the incorporation of PA in international guidelines of the World Health Organization (i.e., 60 minutes of daily moderate to vigorously intense PA) [1]. Despite the well-known benefits, about half of the children in the U.S. and the Netherlands do not meet this guideline [2,3].

Established correlates of children’s PA behavior are male gender, PA enjoyment/preferences, and peer support [4-9]. In addition, increasing evidence suggests that attributes of the perceived physical environment such as functionality, traffic safety, attractiveness, and accessibility are also associated with PA [10-13]. Evidence for this relationship in children is however mixed [11,13]. This mixed evidence is greatly influenced by differences in the measurement of attributes of the physical environment and PA (objective versus subjective) and a lack of systematic investigation of moderators of environmental influences [14]. In addition, several PA domains (e.g., outside play, organized sports, active transport) may have different environmental correlates (e.g., outside play is conceptually matched to playgrounds rather than active transport). Conceptual mismatching of attributes of the physical environment to specific PA domains may be another reason for the mixed evidence in children [15,16].

Outside play (i.e., PA without any given tasks or goals; unstructured free play) is such a PA domain, that has been recommended as most appropriate to increase PA in young children [17]. Outside play has been shown to contribute substantially to children’s total PA levels [12,18-23], also in different specific contexts such as school grounds, sports facilities, urban green space and active transport [24]. In addition, outside play is positively associated with children’s social skills as they learn to account for each other [25-27] and provides challenges that foster the development of new motor skills in a self-regulatory way [28]. In order to promote outside play effectively, the determinants of this behavior should be examined. Three studies have examined correlates of outside play duration related to the physical and social environment, and have reported that the family environment (e.g., parental rules, parental attitudes regarding outside play) was the strongest construct of variables related to outside play, and that the perceived physical environment was considered promising in fostering PA, but they explained only a small proportion of outside play [29-31].

Based on several conceptual ecological frameworks and an umbrella review of Ding and colleagues^12-15^, it is recommended to include potential moderators in the investigation of the relationship between physical environment and PA behavior [14]. The perceived physical environment can directly influence children’s outside play behavior, but the strength of this relationship may depend on activators/inhibitors of the social environment. More specifically, parents may play a crucial role in a child’s relative exposure to the perceived physical environment, and thus also on outside play. Investigating the moderating influence of the social environment on the relationship between the perceived physical environment and outside play is thus a crucial next step in understanding the mechanisms that underlie outside play. Based on results from other studies, some variables are of special interest. First, as young children’s exposure to the neighborhood are considered relatively dependent on their parents, factors such as negative parental attitudes, worry or restrictions may attenuate the relationship between the perceived physical environment and outside play [14,32,33]. Second, factors such as social capital in the neighborhood may strengthen the perceived physical environment – outside play relationship. Neighborhoods with high social capital may be able to reinforce social norms about the plural benefits of PA, and via these social norms – may have increased perceived safety in places where children are likely to regularly participate in outside play [34,35]. In addition, parents experiencing social cohesion may grant their child more autonomy in following up on their needs to play outside.

Consequently, the present study addresses the question to what extent the factors of the social environment – expressed as parenting influences and social capital - moderate the relationship between the parent-perceived physical environment and outside play in five and seven year-old children.

## Methods

### Study design and participants

This study is embedded in the KOALA Birth Cohort Study that follows a group of (originally) 2834 children of healthy pregnant women from the general population who participated in an ongoing prospective cohort study on pregnancy-related pelvic girdle pain. The study addresses two major themes; allergy/asthma, and growth/development. Children are located mainly in the south of the Netherlands, in municipalities of various sizes, including a variety of spatial settings [36]. From the total KOALA-cohort (*N* = 2834), 1875 parents provided information on their child’s outside play around five years, and 1525 reported on their child’s outside play around age seven years. As the perceived physical environment may not be longer valid for the second measurement when participants have moved, we omitted participants’ measurements at follow up when moved home between baseline and follow-up (n = 208, 13.6%). Consequently, follow-up of outside play was available for 1317 participants. All parents gave written informed consent. The present study was approved by the Medical Ethics Committee of Maastricht University Medical Center+.

### Perceived physical environment

Around child age of five years, parents completed a 48 items questionnaire assessing characteristics of the neighborhood environment. Perceived social as well as physical aspects of the environment were assessed through self-completed questionnaires. The construction of the questionnaire was based on the Neighborhood Environment Walkability Scale but was modified to reflect the Dutch built environment, including items relevant to children (e.g., playgrounds, school yards, and dog waste) [37]. A full description and reliability statistics of all scales can be found in the Additional file 1: Table S1. Response scales were constructed according to level of agreement with statements like “Most streets in the neighborhood have cycle paths” or “The neighborhood is a real community”, and consist of five-point scales ranging from “I strongly disagree” to “I strongly agree”. Composite variables were created from individual items by the inspection of Cronbach’s alpha (between 0.70 and 0.80) and principal component analyses for potential constructs with a Cronbach’s alpha of at least 0.60 (Schmidt S, Sleddens E, de Vries S, Gubbels J, Thijs C: The relationship between the neighborhood and body weight development in children: the KOALA Birth Cohort Study, submitted). The following constructs of the perceived physical environment were identified: accessibility (6-item sum score), functionality (6-item sum score), attractiveness (7-item sum score), satisfaction (3-item sum score), and traffic safety (4-item sum score). The use of each of these constructs is recognized in studies investigating the supportiveness of the environment for PA [11,38,39].

### Social environment

Factors of the social environment were expressed as 1) parenting influences and 2) social capital. Parenting influences were assessed by a set of nine questions, based on the Dutch translation of the Child Feeding Questionnaire (CFQ) [40]. The nutrition-related items of the CFQ were translated into a PA-related parenting questionnaire [41]. Parenting influences were defined as the 1) perceived influence of the respondent and their partner on the PA behavior of their child (both 3-item sum scores), 2) attitude towards child PA (5-item sum score), 3) perceived responsibility regarding child PA (2-item sum score), 4) concern regarding child PA (3-item sum score), 5) restriction of child screen time (6-item sum score), 6) pressure towards child to be active (3-item sum score), and 7) monitoring of child PA (2-item sum score). Average reliability (Cronbach’s alpha) of these scales was 0.67, with a range of 0.57 – 0.93. A complete description and reliability statistic of these scales is presented in the Additional file 1: Table S1.

Social capital was assessed by a set of five items, based on earlier empirical research on the influence of social capital on obesity and PA [42]. Cronbach’s alpha for this scale was 0.87 (see Additional file 1: Table S1).

### Outside play

Outside play was defined as the total duration of unstructured outside play in an average week, without organized sports, school physical education, and active transport. Outside play was assessed by questionnaire, both at child age five and seven years using an identical set of questions. First, parents were asked on how many days their child played outside in an average week for the last four weeks. The eight response categories ranged from “never or less than one day on average” till “seven days per week”. Second, parents were asked to indicate the average duration of outside play. The five response categories were: shorter than half an hour (computed as 15 minutes), half to one hour (computed as 45 minutes), one to two hours (computed as 90 minutes), two to three hours (computed as 150 minutes) and three hours or more (computed as 210 minutes). The frequency and duration of child outside play were multiplied to arrive at an average minutes of outside play per week. The date of completing the questionnaire was used to classify the season (i.e., winter, autumn, spring, and summer).

### Statistical analyses

We first evaluated the association among outside play and all attributes of the perceived physical environment, and the social environment (parenting influences and social capital). To do this, we performed repeated measures linear mixed model analyses with outside play at age five and seven years as the dependent variable. We entered season of outside play measurement at age five and seven as a random factor. By doing so, we allowed each child to have its own random slope for season-combinations at five and seven years, while using an autoregressive (AR1) covariance structure. We examined all analyses using the following sequence: model 1) factor only adjusted for covariates (i.e., gender, maternal education, and child age); model 2) factor adjusted for covariates and all variables of their block (i.e., perceived physical environment versus parenting influences and social capital); 3) factor adjusted for covariates and all above described variables of the perceived physical environment, parenting influences and social capital (final model).

We tested for moderation by entering interaction terms between each of the perceived physical environment variables, and each of the parenting influences and social capital variables, using the same repeated measures linear mixed model analyses as described above, with interaction terms for the moderators. We examined all potential moderating associations using the following sequence: model 1) interaction term only adjusted for main effects of the interaction and covariates; model 2) as 1, but also adjusted for previously defined statistically significant main effects; model 3) as 2, but also adjusted for previously defined non-significant main effects; 4) as step 3, but also adjusted for other statistically significant interaction terms in the model (final model). Finally, we stratified consistent significant interactions for interpretation purposes, using a median split.

Based on results from previous studies [11,25,43,44], we investigated the potential confounding influence of seasonality (autumn, winter, spring, summer), gender, age of the child, and maternal education. For maternal education, categories were low (no education, primary school, or ≤ 3 years of general secondary school), mid-low (< 3 years of general secondary school), mid-high (higher vocational training, undergraduate programs, or bachelor’s degree), and high (higher academic education) [45]. None of these potential confounders were associated with a change of more than 10% in any of our coefficients after adjustment. However, to improve the precision of our models, these four variables were entered in our models as covariates [46].

To compare the relative strength of associations among variables, we used standardized coefficients in all models. We defined statistically significant moderation as *p* < 0.10 and a statistically significant association for main effects as *p* < 0.05. As we performed various model variations to investigate consistent significant interactions, correction for multiple testing was not applied. All analyses were performed with SPSS version 20.0 (IBM Corp., NY, USA).

## Results

The total study sample consisted of 1875 children (Table 1). As almost all respondents are mothers of the child, the perceived influence of the respondent and partner on outside play is hereafter defined as perceived influence of the mother. In total, 91.1% of the participants had at least a mid-high educational level (i.e., higher vocational training, undergraduate programs, or Bachelor’s degree) [45]. Children had a mean age of 5.0 years (SD = 0.5) at baseline and 7.0 years (SD = 1.2) at follow-up and spent on average approximately 60 more minutes in outside play per week at seven years compared to five years of age (both boys and girls). Boys spent significantly more time in outside play than girls at both five and seven years (p < 0.01). There were significant differences in outside play duration between all seasons, at both five and seven years (p < 0.01 for both five and seven years, data not shown). Mean scores of each scale are presented in the Additional file 1: Table S1.

**Table 1:** **Characteristics of the study population**

	**Total**		**Boys**		**Girls**	
	***N***	**Mean (SD)**	***n***	**Mean (SD)**	***n***	**Mean (SD)**
Age at baseline (years)	1875	5.0 (0.5)	956	5.0 (0.5)	919	5.0 (0.5)
Age at follow-up (years)	1407	7.0 (1.2)	714	7.1 (1.1)	693	7.0 (1.3)
Gender of parent (female)	1830	97.8%	935	97.7%	895	97.7%
Maternal education (missing *n* = 186)						
Low maternal education	4	0.2%	3	0.3%	1	0.1%
Mid-low maternal education	155	8.7%	81	8.9%	74	8.4%
Mid-high maternal education	687	38.4%	336	36.9%	351	39.9%
High maternal education	943	52.7%	490	53.8%	453	51.5%
Parent perceived neighborhood characteristics						
Satisfaction (range 1-5)	1875	3.8 (0.9)	956	3.8 (0.9)	919	3.8 (0.9)
Functionality (range 1-5)	1875	3.0 (0.8)	956	3.0 (0.8)	919	3.0 (0.8)
Traffic safety (range 1-5)	1875	3.3 (1.0)	956	3.2 (1,0)	919	3.3 (1.0)
Attractiveness (range 1-5)	1875	3.9 (0.6)	956	3.9 (0.6)	919	3.9 (0.5)
Accessibility (range 0-7)^†^	1875	3.4 (1.5)	956	3.4 (1.5)	919	3.4 (1.5)
Outside play at 5 years^‡^	1875	619.2 (365.4)	956	648.6 (365.4)*	919	588.6 (363.1)*
Outside play at 7 years^‡^	1317	683.5 (347.1)	664	708.8 (344.9)*	653	659.7 (347.9)*

^†^According to the number of facilities (forest, school, playground, playing field, (unpaved) gym or exercise facility, swimming pool) accessible for physical activity within 10 minutes of walking. ^‡^presented as minutes per week. *difference = p < 0.01.

We investigated dropout differences between baseline and follow-up regarding all variables presented in Table 1 and Additional file 1: Table S1. Respondents who dropped out were solely somewhat less restrictive of sedentary behaviors (mean score 3.02 versus 2.93; *p* = 0.03), and had a somewhat older child (4.99 versus 5.08 years; *p* = 0.01). At baseline, 97.8% of the respondents that filled in the questionnaire were mothers. Unfortunately we did not assess respondent’s gender at follow-up, and thus we were unable to account for potential differences in subsequent analyses. However, because in previous and subsequent annual questionnaires of the KOALA Birth Cohort the percentage of mothers were all above 95%, one can confidently assume that the percentage mothers at our specific follow-up measurement was also above 95%.

### Main effects of potential predictors of outside play

Perceived attractiveness of the neighborhood and accessibility of PA facilities were related to more (minutes of) outside play over a follow-up period of approximately two years (Table 2, model 1-3). When adjusted for parenting influences and social capital however, the association with attractiveness was attenuated (Table 2, final model). In contrast, accessibility remained statistically significant in all models, implying that outside play was associated with better accessibility of PA-related places within 10 minutes walking distance of home, independent of all variables presented in Table 2.

**Table 2:** **Associations among the perceived physical environment, parenting influences, social capital and child outside play development between five (** ***n*** **= 1875) and seven years (** ***n*** **= 1317)**

	**Model 1 std. beta (95% C.I.)**	**Model 2 std. beta (95% C.I.)**	**Model 2 std. beta (95% C.I.)**	**Final model std. beta (95% C.I.)**
Functionality	−0.01 (−0.05 to 0.03)	−0.03 (−0.07 to 0.01)		−0.03 (−0.07 to 0.01)
Traffic safety	0.07 (0.03 to 0.11)**	0.04 (−0.01 to 0.08)		0.03 (−0.02 to 0.07)
Attractiveness	0.07 (0.03 to 0.12)**	0.06 (0.02 to 0.10)*		0.01 (−0.04 to 0.05)
Accessibility	0.06 (0.02 to 0.10)**	0.06 (0.02 to 0.11)**		0.05 (0.01 to 0.09)*
Attitude	0.13 (0.09 to 0.17)**		0.09 (0.05 to 0.13)**	0.09 (0.05 to 0.13)**
Perceived responsibility	0.002 (−0.04 to 0.04)		0.01 (−0.03 to 0.05)	0.01 (−0.04 to 0.05)
Concern	−0.13 (−0.17 to −0.09)**		−0.04 (−0.09 to −0.01)*	−0.04 (−0.09 to −0.001)*
Restriction	−0.24 (−0.28 to −0.20)**		−0.22 (−0.26 to −0.17)**	−0.21 (−0.26 to −0.17)**
Pressure	0.06 (0.02 to 0.10)**		0.04 (−0.002 to 0.08)	0.04 (−0.01 to 0.08)
Monitoring	−0.02 (−0.06 to 0.02)		−0.01 (−0.05 to 0.03)	−0.01 (−0.05 to 0.03)
Social capital	0.12 (0.08 to 0.16)**		0.08 (0.04 to 0.12)**	0.07 (0.03 to 0.11)**

Standardized coefficients from repeated measures linear mixed models. Dependent variable is outside play at five and seven years. Season at five and seven was entered as random factor, allowing each respondent to have its own random slope for season-combinations, using an autoregressive covariance structure. Model 1 is only adjusted for covariates (gender, maternal education, and child age). Model 2 is adjusted for covariates and all variables in their block (second and third column). Final model is adjusted for covariates and all variables in the table. *p < 0.05, **p < 0.01.

A positive parental attitude towards child PA was related to more outside play; independent of other parenting influences and factors of the perceived physical environment (Table 2). Social capital was also independently related to more outside play. By contrast, restriction of screen time and parental concern regarding child PA were independently related to less outside play. Restriction of screen time showed the strongest association with outside play of all variables examined: parents reported significant less outside play if they thought that they needed to actively restrict their child’s screen time. Again, this association was independent of variables presented in Table 2.

### Moderating relationships between potential predictors of outside play

Two combinations of variables demonstrated moderation between the perceived physical environment, parenting influences and social capital in the amount of outside play at age five and seven (Table 3, model 1-4).

**Table 3:** **Moderators of the longitudinal relationship between the perceived physical environment and child outside play between five (** ***n*** **= 1875) and seven years (** ***n*** **= 1317)**

**Interaction terms**	**Model 1 std. beta (90% C.I.)**	**Model 2 std. beta (90% C.I.)**	**Model 3 std. beta (90% C.I.)**	**Final model std. beta (90% C.I.)**
Functionality * perceived responsibility	**0.034 (0.001 to 0.063)°**	0.038 (0.008 to 0.068)*	0.041 (0.011 to 0.070)*	**0.035 (0.004 to 0.066)°**
Traffic safety * perceived responsibility	0.031 (−0.019 to 0.064)	**0.033 (0.001 to 0.065)°**	0.039 (0.005 to 0.070)*	0.031 (−0.002 to 0.064)

Standardized coefficients from repeated measures linear mixed models. Dependent variable is outside play at five and seven years. Season at five and seven was entered as random factor, allowing each respondent to have its own random slope for season-combinations, using an autoregressive covariance structure.

Model 1 is only adjusted for covariates (gender, maternal education, and child age) and main effects of the interaction. Model 2 is adjusted for covariates significant main effects of Table 2 (accessibility, attitude, concern, restriction, social capital). Model 3 contains covariates and all main effects of Table 2. Final model is equal to Model 2, but interactions are also adjusted for each other. **°**p < 0.10, *p < 0.05.

Perceived responsibility moderated the perceived environmental influence of functionality on outside play, consistently across all models (Table 3). When stratified, children from parents with high responsibility regarding their child’s outside play demonstrated that functionality was related with more outside play (0.04, 95% C.I. = −0.07 to 0.15), while among parents with low responsibility, functionality was related with a less outside play (-0.03, 95% C.I. = −0.09 to 0.04).

Next to functionality also traffic safety showed interaction with perceived responsibility, but this only appeared after adjustment for main effects (Model 3) and slightly attenuated after adjustment for the other interactions (Model 4). Stratification showed that high responsibility strengthened the association between traffic safety and outside play (0.10, 95% C.I. = −0.03 to 0.23) versus (standardized beta 0.06, 95% C.I. = −0.003 to 0.12). Note that stratified models are adjusted for child age, child gender, and maternal education. In both strata, main effects were not statistically significant.

## Discussion

This study has investigated the extent to which the social environment – expressed as parenting influences and social capital - moderated the relationship between the perceived physical environment and the development of children’s outside play between five and seven years. We have showed that accessibility of PA facilities, positive parental attitude towards PA and social capital were associated with more outside play, while parental concern with respect to child PA participation and especially restriction of screen time were related with less outside play.

Although we only found a limited number of relatively weak interactions, this study demonstrated that the impact of the perceived physical environment might differ across levels of parenting responsibility. More specifically, this study showed that among children with parents with high responsibility towards their child PA, functionality of the neighborhood was related to more outside play; while in children with parents with low responsibility towards their child PA level, functionality was related to less outside play. The latter may reflect that functionality in the present study may be merely associated with a relative paucity of non-predefined public open space where children can play outside. This is supported by qualitative evidence that the usage of public open space depend on the child needing to cross busy roads [47]. On the other hand, parents who feel responsible for the amount of their child PA may deliberately provide their child with the autonomy to play outside at spaces that they think are appropriate and safe.

One study specifically reported on the fairly minor influence of the perceived physical environment on outside play, and reported that the social environment overpowered the perceived physical environment in explaining outside play behavior [31]. This is, to some extent, in line with the results of our study, which showed that the influence of the attractiveness of the neighborhood was attenuated when adjusted for parenting influences and social capital. In addition, previous research supports our finding that parental attitude is an important predictor of a higher amount of child outside play [33,48]. The evidence for the importance of social capital on child PA and especially outside play is scarce. It however seems plausible that social capital directly influences child outside play via the availability of more social contacts in the neighborhood, and indirectly influences child outside play by decreasing parental worries related to social safety (‘stranger danger’).

Parental restriction of screen time was strongly associated with less outside play at age five and seven. This seems in contrast to one previous study that highlighted the importance of parental rules in regulation of outside play [31]. However, in the present study these ‘rules’ have a restrictive character, which may be less suitable for promoting outside play than positively formulated, supportive rules. This resembles previous findings from the KOALA study that parental restriction of sedentary activities was related to more sedentary behavior, whereas parental promotion of PA was associated with more activity [41]. This is also in line with studies regarding child feeding and snacking behavior, which showed that parental restriction of screen time was related with increased child snack consumption, energy intake and body weight [49,50]. In addition, personal factors such as PA enjoyment or peer support from other children may also exert great influence on child outside play, and perhaps even attenuate our associations of interest [4].

Our findings regarding moderation of parenting influences and perceived physical environment in the regulation of outside play behavior are in line with the ecological perspective on energy balance related behaviors [51,52] and PA specifically [53]. However, we only found a limited number of relatively weak interactions. To date, studies on the moderating factors within the perceived physical environment regarding PA remain scarce [14]. The few studies investigating these moderating factors are often incorporated within additional analyses of an intervention study, and mainly focused on socio-demographical factors (i.e., gender, age and race) [14,54], which prohibits a valid comparison of our findings regarding the moderating influences of parenting influences and social capital with other studies. In line with recent advances in the field of parenting influences on child nutrition which reported upon moderations between general parenting style and specific parenting practices [55,56], future studies are encouraged to focus on moderation among social environmental factors, and between the social and the (perceived) physical environment [14]. In addition, attributes of the perceived physical environment may also interact with each other (e.g., relative accessibility of PA places may become less important if they are highly attractive). Based on recent work in the field of moderation of health behaviors, we suggest that social environmental factors (and especially parenting influences) act as a gatekeeper in the exposure of children to the perceived physical environment, and thus moderates its influence on PA and outside play [14,57].

### Strengths and weaknesses

Strengths of the present study are, first, the consistent approach of investigating the relationship among the perceived physical environment, parenting influences, social capital, and outside play. Second, this study addresses the moderating influences of the parenting influences and social capital, which is relatively new in the field of health behaviors. Third, a longitudinal design was used, providing insight into associates and moderating factors regarding outside play development across a two-year period.

This study had some limitations; namely the relatively high educational level in the KOALA Birth Cohort Study [58] may limit generalization of our results with studies that included participants with relatively lower educational levels. Our dropout analyses showed that there was no important selective dropout and that attrition of was not likely to have influenced our results.

We used the meteorological seasons (i.e., autumn, winter, spring, and summer) to adjust for seasonality. However, more precise adjustment for weather-related burdens for children to perform outside play or PA (e.g., rainfall, temperature, and sunshine) may be available. As appropriate adjustment for seasonality is essential in studies investing PA [43], future studies are encouraged to examine the relative influence of these weather conditions on PA and outside play and the role of these weather conditions as a confounder.

The comparability of the standardized beta coefficients across model variations indicates consistency and statistical rigor of our models. We acknowledge that our effect sizes were relatively small. However, these small standardized effect sizes may be underestimated due to the relatively high error variance in the parent reported variables (main variables, moderators as well as outcomes). In addition, by allowing respondents to indicate the duration of outside play in five response categories, some measurement error is introduced and precision is compromised. These errors may be subsequently magnified by the multiplication effect regarding the assessment of the frequency of outside play. We thus encourage future studies to improve the reliability and precision of these parental reports by external validation with recent technological advances, examining test-retest methodologies or asking both the father and mother to report on their child’s outside play independently.

To date, outside play can only be assessed by parent reports, as a distinction between outside play and other types of PA cannot be made by the currently used objective measurements (e.g., accelerometers or heart-rate monitors). However, recent technological advances provide opportunities for combining global positioning system (GPS), Geographic Information Systems (GIS), and accelerometers. One recent study managed to apply these recent methodologies in an objective measurement instrument of outside play, and investigated differences in child gender, age, and comparisons with solely accelerometer-based MVPA [24]. Future studies are encouraged to replicate this methodology and move on to investigate the influence of the (perceived) physical environment and/or social environment on objective outside play behavior.

Regarding the relative contribution of outside play to the total PA, one study recently investigated the relationship between the amount of parent reported outside play and the amount of accelerometer-measured PA. This study reported that more outside play (<1 hour per day versus 1-2 hours per day and >2 hours per day) was related with significantly lower sedentary time and higher PA of both light and moderate-to-vigorous intensity [22]. In addition, only one study has estimated the proportion of strenuous PA during outside play in an intervention promoting outside play using special playgrounds and it has reported that approximately 35% of the time spent in outside play was moderate to vigorously active [59]. Future studies are encouraged to quantify the contribution of outside play, relative to total PA and other PA domains.

It should be noted that this study solely focused on outside play. Therefore, these results are not generalizable to other PA domains such as organized sports and active transport. Outside play cannot be directly associated with the earlier established benefits of PA, such as its relationship with childhood obesity. Nonetheless, considering its multiple benefits such as increased PA [21], motor abilities, and social skills [25-27], outside play arguably contributes to improved childhood health in the long run.

## Additional file

Additional file 1: Table S1. Characteristics of attributes of the perceived physical environment and moderators in the relationship between the perceived physical environment and child outside play.
