# Supplementary material for: Moderators of the longitudinal relationship between the perceived physical environment and outside play in children: the KOALA birth cohort study
Source: Int J Behav Nutr Phys Act. 2014 Dec 12;11:150. doi: 10.1186/s12966-014-0150-8 (PMC4271341; doi:10.1186/s12966-014-0150-8)
Supplement: Additional file 1: Table S1. — Characteristics of attributes of the perceived physical environment and moderators in the relationship between the perceived physical environment and child outside play. [file 12966_2014_150_MOESM1_ESM.doc]

**Additional file 1: Table S1**. Characteristics of attributes of the perceived physical environment and moderators in the relationship between the perceived physical environment and child outside play.

| Construct | | Description / Items | Cronbach’s alpha | Mean (SD) |
| --- | --- | --- | --- | --- |
| Potential moderators | | |  |  |
|  | Attitude | 5-item mean score; usefulness, importance, healthiness, pleasantness, tediousness of child physical activity | 0.77 | 4.5 (0.5) |
|  | Perceived responsibility | 2-item mean score; perceived responsibility regarding child amount of physical activity and limit of physical activity | 0.93 | 3.6 (0.9) |
|  | Concern | 3-item mean score; concern regarding child lack of physical activity, diet for weight management, getting overweight | 0.68 | 1.3 (0.5) |
|  | Restriction | 6-item mean score; if not regulated child would watch too much TV/not enough physical activity, I want to be sure that my child does not watch too much TV/plays too many computer games, I reward good behavior of my child with TV or computer games, I deliberately keep my child away from the TV or computer | 0.59 | 3.0 (0.7) |
|  | Pressure | 4-item mean score; stimulation of PA against child will, parental pressure towards child getting as much active transport as possible, parents make sure that child is sufficiently active | 0.57 | 3.7 (0.6) |
|  | Monitoring | 2-item mean score; parental monitoring of TV/computer games, and physical activity | 0.64 | 3.9 (0.8) |
|  | Social capital | 5-item mean score; willingness to help each other in neighborhood, presence of community, trust in neighborhood, getting along in neighborhood, sharing of norms and values | 0.87 | 3.8 (0.6) |
| Attributes of the perceived physical environment | | |  |  |
|  | Accessibility | 7-item mean score; number of facilities for PA within 10 minutes walking distance from forest, school, playground, playing field (unpaved), gym or facility for exercise, swimming pool | n.a. | 3.4 (0.5) |
|  | Traffic safety | 4-item mean score; speed of traffic (max 30kmph), exhausts, ability of child outside play regarding traffic, compliance with speed limit | 0.74 | 3.3 (1.0) |
|  | Functionality | 6-item mean score; availability of footpaths and stairs, maintenance of footpaths, separation of footpaths from streets, availability of cycling paths, separation of cycle paths from streets, availability of different routes | 0.64 | 3.0 (0.8) |
|  | Attractiveness | 7-item mean score; availability of green, amount of litter, presence of residential blocks, presence of detached houses, presence of abandoned houses, amount of noise, amount of dog feces | 0.66 | 3.9 (0.6) |
|  | Satisfaction | 3-item mean score; satisfaction with opportunities to play outside, satisfaction with opportunities to walk, satisfaction with opportunities to cycle | 0.85 | 3.8 (0.9) |

Note: all constructs have 5-item response scales
